# Toxicity and genotoxicity of imidacloprid in the tadpoles of *Leptodactylus luctator* and *Physalaemus cuvieri* (Anura: Leptodactylidae)

**DOI:** 10.1038/s41598-022-16039-z

**Published:** 2022-07-13

**Authors:** Caroline Garcia Samojeden, Felipe André Pavan, Camila Fátima Rutkoski, Alexandre Folador, Silvia Pricila da Fré, Caroline Müller, Paulo Afonso Hartmann, Marilia Hartmann

**Affiliations:** 1 Ecology and Conservation Laboratory, Federal University of Fronteira Sul, Erechim, RS 99.700-000 Brazil; 2 Aquatic Toxicology Study Center, Regional University of Blumenau, Blumenau, SC 89.012-170 Brazil

**Keywords:** Conservation biology, Environmental sciences

## Abstract

Imidacloprid is a neonicotinoid insecticide used to control agricultural pests around the world. This pesticide can have adverse effects on non-target organisms, especially in aquatic environments. The present study evaluated the toxicity of an imidacloprid-based insecticide in amphibians, using *Leptodactylus luctator* and *Physalaemus cuvieri* tadpoles as study models. Spawning of both species were collected within less than 24 h of oviposition from a non-agricultural land at Erechim, Rio Grande do Sul state, Brazil. Survival, swimming activity, body size, morphological malformations, and genotoxic parameters were analyzed at laboratory conditions. A short-term assay was conducted over 168 h (7 days) with five different concentrations of imidacloprid (3–300 µg L^−1^) being tested. The insecticide did not affect survival, although the tadpoles of both species presented reduced body size, malformed oral and intestine structures, and micronuclei and other erythrocyte nuclear abnormalities following exposure to this imidacloprid-based compound. Exposure also affected swimming activity in *L. luctator*, which reflected the greater sensitivity of *L. luctator* to imidacloprid in comparison with *P. cuvieri*. The swimming activity, body size, and malformations observed in *L. luctator* and the morphological malformations found in *P. cuvieri* indicated that even the lowest tested concentration of the insecticide were harmful to amphibians. At concentrations of over 3 μg L^−1^, *P. cuvieri* presents a smaller body size, and both species are affected by genotoxic cell damage. This demonstrates that imidacloprid is potentially toxic for the two study species at environmentally relevant concentrations.

## Introduction

Given their permeable skin and sensitivity to changes in environmental conditions, amphibians are considered to be excellent bioindicators of environmental quality^1^. As a consequence of this sensitivity, amphibian populations are declining worldwide, and the number of endangered species has grown considerably in recent years^2^. The known causes of amphibian declines are many and complex, and include well-known concerns such as habitat destruction, climate change, a pathogen fungus and widespread use of pesticides^3–5^. Amphibians are particularly sensitive to pollutants because they occupy a transitional niche between terrestrial and aquatic ecosystems^6^. Most species require humid or aquatic environments in which to reproduce, and spawning may often coincide with the periods when pesticides are applied to agricultural settings, in the spring and summer^7^, increasing the susceptibility of these vertebrates to pollutants.

Toxicological studies using native species have been important for the assessment of the sensitivity of amphibians to the effects of toxic substances. *Leptodactylus luctator* (Hudson 1892) and *Physalaemus cuvieri* (Steffen 1815), for example, are native to South America. *Leptodactylus luctator* is widely distributed in South America^8^, while *P. cuvieri* inhabits anthropogenically modified areas^9^ in Brazil, Argentina, and Paraguay. Both species are highly adaptable, occurring in different types of either modified and unmodified habitats. They reproduce preferentially in temporary bodies of water, which are often common in agricultural areas, where they deposit their spawns in large foam nests on the surface of the water^10,11^. Recent studies have reported that both *L. luctator* (appearing as *Leptodactylus latrans*) and *P. cuvieri* are sensitive to exposure to glyphosate, which may provoke developmental, behavioral, and morphological alterations, as well as genotoxic effects and lethality^12^. In addition, the insecticide chlorpyrifos, which alters swimming activity^13^, and the mixture of glyphosate and 2,4-d, have been shown to be toxic to *L. luctator*^14^.

Neonicotinoid insecticides were first marketed in the 1990s, and almost immediately replaced organophosphates and carbamates for the control of herbivorous insects, becoming the most used insecticide class for the control of agricultural pests worldwide since then^15^, with imidacloprid being the most-widely used of all neonicotinoids^16,17^. Imidacloprid was the fourth best-selling insecticide in Brazil in 2019, with 9214.45 tons of active ingredient being marketed^18^. With no use restrictions in Brazil, the domestic sales of imidacloprid have increased by 81% from 2011 (5074.00 ton) and by 16% in comparison with 2014 (7951.43 ton)^19^. While the use of neonicotinoids is a global environmental issue, the dispersal, behavior, and effects of their residues are still very poorly understood, in general^20^.

Imidacloprid is indicated for foliar application on crops such as lettuce, coffee, sugarcane, beans, tobacco, corn, tomatoes, wheat, and grapes^21^. It has a neurotoxic action in insects, interacting chemically to mimic the action of acetylcholine by binding to the nicotinic receptors (nAChRs) of this important neurotransmitter^22^. By acting selectively on insect nAChRs^23^, imidacloprid triggers excessive neuron stimulation, which results in the insect’s death^15^.

Imidacloprid has low sorption and degrades slowly in the soil, which allows it to leach into the groundwater or reach surface water through runoff or leaching^20,24^. Once in the water, imidacloprid can be persistent, with a half-life of 30 days and low biodegradability^25^. Imidacloprid is often detected in surface water^16,26^, and has been one of the insecticides most detected in drinking water in Brazil over the past 10 years^27^.

Some countries have established legal limits for imidacloprid concentrations in water sources. In Canada, for example, the maximum permitted concentration of chronic exposure of imidacloprid for the protection of aquatic life is 0.23 μg L^−1^^28^, while in the Netherlands, the environmental risk index of acute toxicity for aquatic organisms is 0.2 μg L^−1^ and 0.0083 μg L^−1^ for chronic toxicity^29^. In the last few years, imidacloprid has been banned for use in open plantations in the United Kingdom and European Union^30^. In Brazil, however, the only restriction imposed up to now has been a limit of 300 µg L^−1^ for drinking water in Rio Grande do Sul state^31^.

There are several studies about of imidacloprid toxicity in many non-target organisms found in aquatic environments, such as aquatic insects^32^, fish^33^, and anuran amphibians^34,35^. In the US Environmental Protection Agency’s measured concentrations of pesticides for aquatic life and human health benchmarks^36^, chronic toxicity values for freshwater invertebrates (0.39 μg L^−1^) and fish (9000 μg L^−1^) were available, but not amphibians. Insects are expected to be more sensitive to imidacloprid than fish and amphibians, but it is not yet known what concentrations actually cause toxic effects on aquatic vertebrates. In fish, there is data showing that several malformations of common carp embryos and larvae were induced by the toxicity of imidacloprid at concentrations of 300 and 1000 µg L^−1^^33^. In anuran amphibians, Feng et al.^37^ observed an increasing in *Rana limnocharis* and *Rana nigronaculata* mortality from imidacloprid doses of 30 mg L^−1^ and 45 mg L^−1^, respectively. However, *R. nigronaculata* showed DNA damage and increased MNs from doses of 0.05 mg L^−1^ and 8 mg L^−1^, respectively^37^. Damage cell and DNA damage^34^ and increased frequency of MNs^38^ were also reported to *Boana pulchella* (denominated *Hypsiboas pulchellus* in the studies) after 96 h exposure to imidacloprid-based insecticide at concentrations of 25 mg L^−1^ and 15 mg L^−1^, respectively. While an insecticide based on imidacloprid and lambda-cyhalothrin caused 100% morality of *Amietophrynus regularis* at doses from 0.05 mg L^−1^^39^ Low levels (e.g., environmental levels^40–42^) have seldom been studied in amphibians. A recent study found that the application of a halfway stimulus in *Rana sylvatica* exposed to low doses of imidacloprid (10 µg L^−1^) demonstrated that the larvae swam shorter distances and spent less time swimmin﻿g^43^, suggesting that imidacloprid exposure may delay the reaction, potentially increasing the risk of ﻿predation^43^.

Given these findings, the present study investigated the toxicity of different concentrations of an imidacloprid-based insecticide in the tadpoles of *L. luctator* and *P. cuvieri* by assessing survival, swimming activity, body size, morphological malformations, and parameters of genotoxicity.

## Results

Exposure to the imidacloprid-based insecticide did not have a significant impact on the survival of the tadpoles of either *L. luctator* and *P. cuvieri* after 168 h. While the mean survival of *L. luctator* was 84.67% (F_5,12_ = 1.16; *p* = 0.380), all (100%) of the exposed *P. cuvieri* tadpoles survived in all the treatments. The data are presented in the supplementary material (Supplementary Table S1 Online).

### Body size

**Figure 1:**
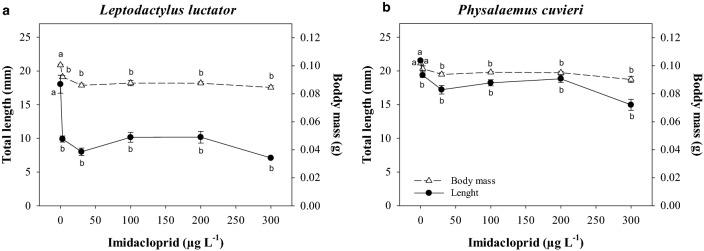
Total length (mm, open triangle) and body mass (g, closed circle) of the *Leptodactylus luctator* (**a**) and *Physalaemus cuvieri* (**b**) tadpoles exposed for 168 h to different concentrations of an imidacloprid-based insecticide. The data are presented as the mean ± SEM (*n* = 3).

Following exposure to imidacloprid, the tadpoles of both species had smaller body sizes than their respective controls (Fig. 1; Supplementary Table S1 Online). The *L. luctator* tadpoles exposed to imidacloprid were 12.9% shorter, on average, than the controls (F_5,12_ = 23.58; *p* < 0.0001) and weighed 49.7% less (F_5,12_ = 25.11; *p* < 0.0001), while the exposed *P. cuvieri* tadpoles were 7.2% shorter (F_5,12_ = 7.74; *p* = 0.0018) and weighed 17.6% less (F_5,12_ = 16.75; *p* < 0.0001) than the control tadpoles.

### Morphological malformations

**Figure 2:**
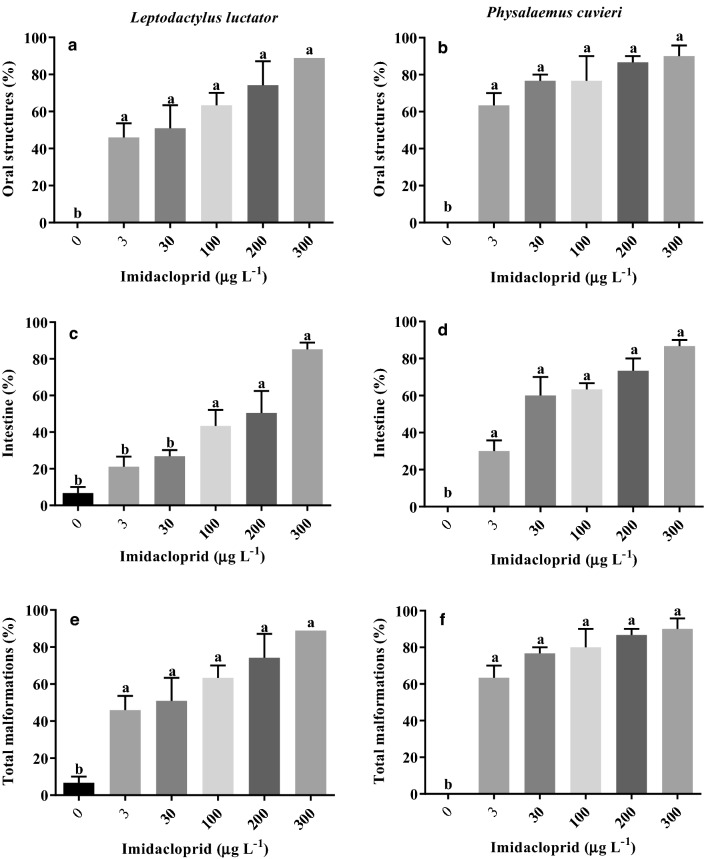
Percentage of occurrence of malformations in the oral structures, intestine, and total malformations (oral structures + intestine) in the tadpoles of *Leptodactylus luctator* (**a**, **c**, **e**) and *Physalaemus cuvieri* (**b**, **d**, **f**) exposed to different concentrations of an imidacloprid based-insecticide for 168 h. The bars represent the mean ± SEM (n = 3). Different letters above pairs of columns indicate significantly different results according to Dunnett’s test (p < 0.05).

**Figure 3:**
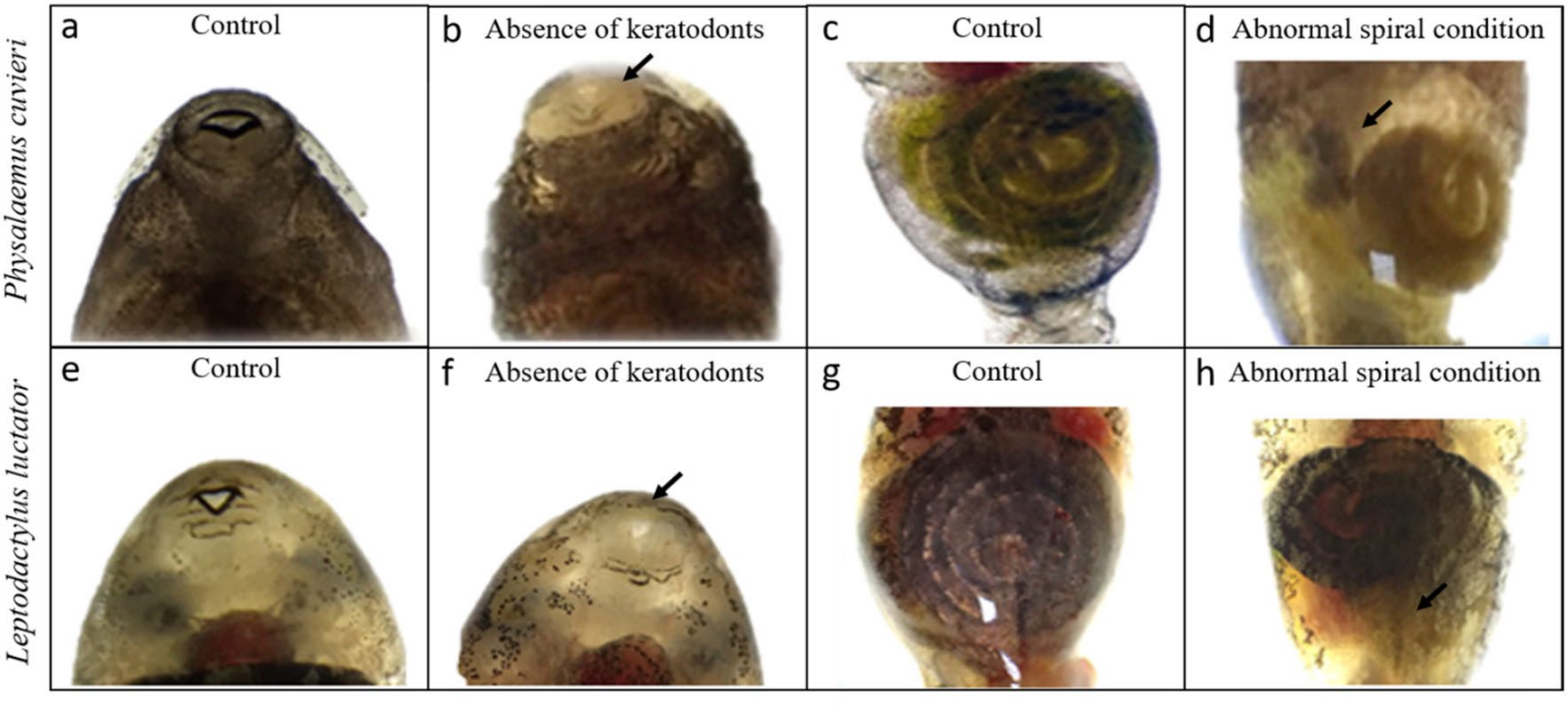
Tadpoles of *Leptodactylus luctator* (**a**–**d**) and *Physalaemus cuvieri* (**e**–**h**). These tadpoles represent the control group (**a**, **c** and **e**, **g**), and individuals exposed to different concentrations of an imidacloprid-based insecticide for 168 h: (**b**) 200 µg L^−1^, malformation of the oral structures; (**d**) 200 µg L^−1^, intestinal malformation; (**f**) 300 µg L^−1^, malformation of the oral structures; (**h**) 300 µg L^−1^ intestinal malformation. (For the color version of figure, the reader is referred to the web version of this article).

Malformation of the oral structures were observed in the *L. luctator* (F_5,12_ = 13.22; *p* = 0.0002) and *P. cuvieri* tadpoles (F_5,12_ = 24.16; *p* < 0.0001) at the lowest concentration (3 µg L^−1^) tested, reaching almost 90% of the individuals exposed to 300 µg L^−1^ of imidacloprid for 168 h (Figs. 2, 3; Supplementary Table S2 Online). Intestinal malformations appeared in *L. luctator* (F_5,12_ = 15.88; *p* < 0.0001) at 100 µg L^−1^ (43.3% of the individuals), reaching 85.2% of the tadpoles exposed to 300 µg L^−1^. All concentrations tested caused significant intestinal malformations in *P. cuvieri* (F_5,12_ = 30.22; *p* < 0.0001; Fig. 2c,d). When tadpoles with oral and intestinal malformations were added together, the total number of malformations showed that 45.9% and 63.3% of the tadpoles exposed to 3 µg L^−1^ of imidacloprid for 168 h presented malformations in both *L. luctator* (F_5,12_ = 11.02; *p* = 0.0004) and *P. cuvieri* (F_5,12_ = 34.05; *p* < 0.0001, Fig. 2e,f; Supplementary Table S2 Online), respectively, with this percentage rising to 90% at 300 µg L^−1^.

### Swimming activity

**Figure 4:**
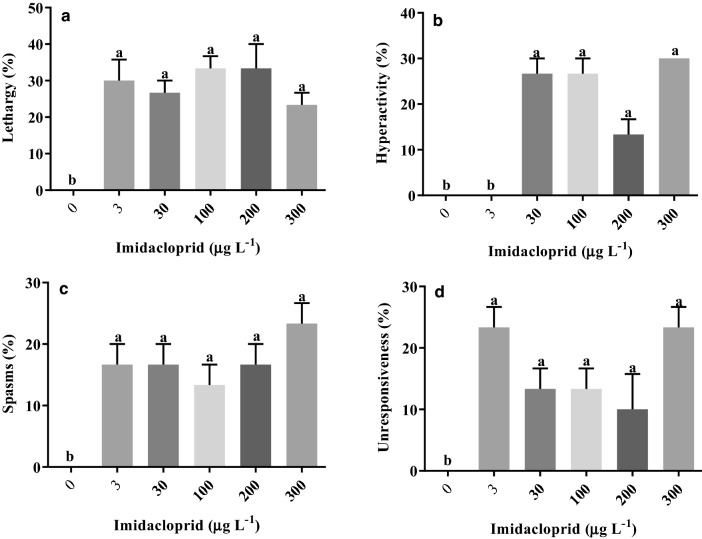
Frequency of lethargy (**a**), hyperactivity (**b**), spasm (**c**), and unresponsiveness (**d**) in *Leptodactylus luctator* tadpoles exposed to an imidacloprid-based insecticide for 168 h. The bars represent the mean ± SEM (*n* = 3). Different letters above pairs of columns indicate significantly different results according to Dunnett’s test (*p* < 0.05).

Exposure to imidacloprid caused changes in tadpole swimming activity in comparison with the control in *L. luctator* only (Supplementary Table S3 Online). The most frequent behavioral alteration was lethargy (30.7% of the exposed tadpoles), followed by hyperactivity (20.7%), and spasms (18.7%). Almost a fifth (18%) of the treated tadpoles were unresponsive (Fig. 4).

In *L. luctator*, lethargy was significantly more frequent in all the imidacloprid treatments in comparison with the control (F_5,12_ = 8.56; *p* = 0.0012) (Fig. 4). Hyperactivity was also significantly more frequent in all treatments at concentrations above 30 µg L^−1^ (F_5,12_ = 33.93; *p* < 0.0001), whereas spasms only increased significantly at the highest concentration (300 µg L^−1^) (F_5,12_ = 5.971, *p* = 0.0053). Unresponsive tadpoles were recorded in all treatments (F_5,12_ = 6.56; *p* = 0.0037).

### Micronuclei (MN) and other erythrocytic nuclear abnormalities (ENAs)

The frequency of micronuclei was significantly higher in the *L. luctator* tadpoles exposed to 200 µg L^−1^ and 300 µg L^−1^ of imidacloprid in comparison with the controls (F_5,12_ = 6.954; *p* = 0.0029; Table 1), and in the *P. cuvieri* tadpoles exposed to the highest concentration (300 µg L^−1^) (F_5,12_ = 8.430; *p* = 0.0013; Table 1), in comparison with their corresponding controls. Other ENAs was significantly higher than the controls at concentrations equal to or above 30 µg L^−1^ in both *L. lucactor* (F_5,12_ = 13.04; *p* = 0.0002) and *P. cuvieri* (F_5,12_ = 11.72; *p* = 0.0003) (Table 1).

**Table 1:** Frequency of micronuclei (MN, %), Erythrocyte Nuclear Abnormalities (ENAs, %), apoptosis (AP, ‰), nuclear bubbles/buds (NB, ‰), binucleated cells (BC, ‰), notched nuclei (NN, ‰), and lobed nuclei (LN, ‰) in the *Leptodactylus luctator* and *Physalaemus cuvieri* tadpoles exposed to different concentrations of imidacloprid for 168 h.

Species	Imidaclorid (µg L^−1^)	MN (%)	Total ENAs (%)	ENAs (per 1000 cells)				
				AP	NB	BC	NN	LN
*Leptodactylus luctator*	0	0b	4.6 ± 0.34 (3–7)b	0b	0.9 ± 0.07 (0–1)b	0.2 ± 0.09 (0–1)b	1.8 ± 0.25 (0–4)b	1.7 ± 0.02 (1–2)b
	3	0.4 ± 0.11 (0–2)b	14.8 ± 4.24 (4–39)b	0.8 ± 0.25 (0–3)b	2.3 ± 1.06 (0–7)b	1.3 ± 0.29 (0–4)b	4.8 ± 1.01 (2–13)b	4.8 ± 1.51 (0–12)b
	30	0.5 ± 0.25 (0–1)b	25.5 ± 4.17 (15–36)a	0.7 ± 0.33 (0–3)b	3.0 ± 0.60 (0–5)b	1.3 ± 0.67 (0–4)b	9.4 ± 1.62 (5–17)a	9.7 ± 1.22 (6–13)a
	100	1.2 ± 0.72 (0–4)b	35.4 ± 2.50 (24–50)a	1.3 ± 0.67 (0–5)b	3.6 ± 1.17 (0–9)b	2.4 ± 1.13 (0–6)b	13.4 ± 0.17 (6–20)a	12.1 ± 0.21 (8–16)a
	200	2.3 ± 0.17 (0–5)a	38.2 ± 1.83 (22–60)a	0.7 ± 0.27 (0–2)b	4.0 ± 0.60 (0–10)b	2.4 ± 0.57 (0–6)b	14.9 ± 0.80 (6–21)a	13.0 ± 0.89 (7–17)a
	300	2.5 ± 0.32 (0–5)a	46.1 ± 6.22 (23–81)a	2.0 ± 0.28 (0–4)a	5.4 ± 1.37 (0–16)a	3.1 ± 0.18 (0–9)a	17.8 ± 2.93 (6–27)a	15.2 ± 1.76 (3–22)a
*Physalaemus cuvieri*	0	0b	2.1 ± 0.55 (0–5)b	0b	0.1 ± 0.07 (0–1)b	0b	1.3 ± 0.22 (0–5)b	0.6 ± 0.19 (0–3)b
	3	0b	15.3 ± 1.93 (5–25)b	0.2 ± 0.19 (0–2)b	1.1 ± 0.07 (0–2)b	0.5 ± 0.08 (0–2)b	5.2 ± 0.29 (1–10)b	6.6 ± 0.99 (0–10)b
	30	0.4 ± 0.11 (0–1)b	23.8 ± 0.68 (14–38)a	0.4 ± 0.17 (0–1)b	2.6 ± 0.76 (0–5)b	1.4 ± 0.25 (0–5)b	8.1 ± 0.37 (3–13)a	9.4 ± 0.53 (3–17)b
	100	0.6 ± 0.26 (0–2)b	27.9 ± 6.56 (13–59)a	0.2 ± 0.14 (0–1)b	3.4 ± 1.34 (0–10)b	1.0 ± 0.00 (0–2)b	9.4 ± 1.11 (6–18)a	13.6 ± 4.02 (5–36)a
	200	0.7 ± 0.19 (0–2)b	35.4 ± 4.16 (23–62)a	0.6 ± 0.05 (0–2)b	6.0 ± 0.79 (1–15)a	2.2 ± 0.54 (0–5)a	12.8 ± 0.98 (9–19)a	12.9 ± 1.80 (6–28)a
	300	1.5 ± 0.21 (0–6)a	43.0 ± 4.09 (12–76)a	0.7 ± 0.13 (0–1)a	7.3 ± 2.16 (0–16)a	2.2 ± 0.51 (0–6)a	13.9 ± 1.06 (4–20)a	17.9 ± 1.11 (3–48)a

The values are given as the mean ± SEM (minimum–maximum ENAs observed in 1000 cells; *n* = 3). Pairs of values followed by different letters in the same line are significantly different according to Dunnett’s test (*p* < 0.05).

**Figure 5:**
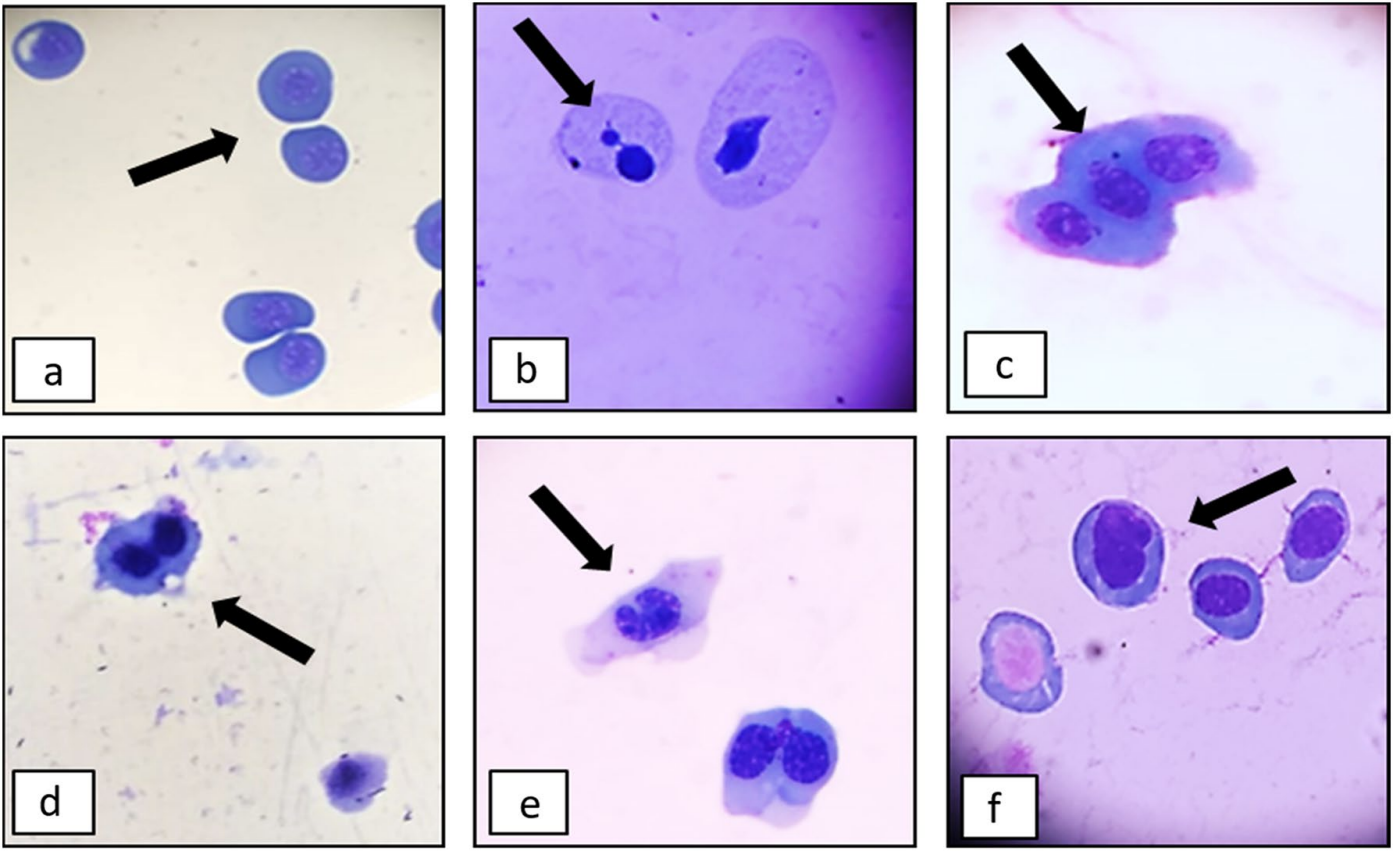
Erythrocyte Nuclear Abnormalities found in the tadpoles of *Leptodactylus luctator* and *Physalaemus cuvieri* exposed to different concentrations of imidacloprid for 168 h. (**a**) Normal cells, (**b**) Micronucleus (MN); (**c**) Nuclear bubble/bud (NB); (**d**) Binucleated cell (BC); (**e**) Notched nucleus (NN); (**f**) Lobed nucleus (LN). (For the color version of Fig. 5, the reader is referred to the web version of this article).

Karyolysis was observed occasionally only in *L. lucactor* (F_5,12_ = 1.709; *p* = 0.2070), however, it was not at a level significantly greater than the control (data not shown). All other types of ENAs (i.e., apoptosis (*L. lucactor*: F_5,12_ = 2.579; *p* = 0.0218; *P. cuvieri*: F_5,12_ = 3.204; *p* = 0.0457), nuclear buds (*L. lucactor*: F_5,12_ = 2.069; *p* = 0. 0475; *P. cuvieri:* F_5,12_ = 4.525; *p* = 0.0150), binucleated cells (*L. lucactor*: F_5,12_ = 2.244; *p* = 0.0468; *P. cuvieri*: F_5,12_ = 5.864; *p* = 0.0057), and notched and lobed nuclei) were all found in both species, predominantly at the highest concentration (Fig. 5, Table 1). Notched (*L. lucactor*: F_5,12_ = 13.21; *p* = 0.0002; *P. cuvieri*: F_5,12_ = 28.25; *p* < 0.0001) and lobed nuclei (*L. lucactor*: F_5,12_ = 15.61; *p* < 0.0001; *P. cuvieri*: F_5,12_ = 7.692; *p* = 0.0019) were the most frequent ENAs, and were significantly more frequent in the 30 µg L^−1^ imidacloprid treatment than in the controls (Fig. 5, Table 1).

## Discussion

Exposure to imidacloprid caused morphological and genotoxic changes in the tadpoles of both *L. luctator* and *P. cuvieri*, although it did not affect survival. This was expected from the concentrations used in the present study (3–300 µg L^−1^), which were mostly below the LC_50_ thresholds (82–366 mg L^−1^) reported for other amphibian species (reviewed by Gibbons﻿ et al.^44^). The imidacloprid concentrations used in this study may nevertheless be representative of the levels of contamination found typically in surface water in agricultural areas^45^, and appear not to cause mortality but showed different chronic effects in other aquatic vertebrate species, such as *R. sylvatica*, exposed to 10–500 µg L^−1^ (cited as ppm) of the insecticide^43^, and the fish *Pimephales promelas* ﻿(10 µg L−1)^46^.

Changes in the development of the tadpoles were manifested by the reduced length and body mass observed in exposed tadpoles of both species, with *L. luctator* being more sensitive to imidacloprid than *P. cuvieri.* Under stress, such as the presence of contaminants, efforts to tolerate the presence of pesticides may compromise an individual’s metabolism and growth^47^. Reduced development of the tadpoles may make them more vulnerable to predation in natural environments, because of both their smaller size (e.g., Carlson and Langkilde^48^) and their reduced physical capacity. Although body size is a highly variable characteristic, there is a general correlation between the size of the tadpoles and the adults^49^. Previous studies have shown that tadpoles with reduced body size may develop into smaller adults, with lower rates of survival and reproductive success^50^. *Leptodactylus luctator* (denominated *L. latrans* in some previous studies) is a relatively large-bodied amphibian, which is important for the defense of the eggs and tadpoles, as well as the avoidance of predation^51^. Morphological changes in the tadpoles might also alter their eventual reproductive success, given that smaller females of both study species are known to be less fecund^52–54^.

Increasing concentrations of imidacloprid caused malformations of the oral and intestinal structures of the tadpoles. The malformation of oral structures may restrict the growth of the individual and differences in tadpoles’ oral morphology may affect its capacity to acquire food^55,56^. The oral structures of these tadpoles consist of labial teeth that are used as food scrapers, and changes in these structures may affect the ability of the tadpoles to forage^55,57,58^. Inefficient feeding may impact growth rates and the accumulation of body mass﻿^57,59,60^, as well as increase the susceptibility of the individual to predation^55,61^. The structural integrity of the intestine is also important to guarantee efficient nutrient absorption and growth^62,63^.

Malformed individuals generally constitute a small proportion of natural amphibian populations, typically less than 2%^64^. In the present study, however, more than 50% of the individuals presented morphological malformations, reflecting the toxic effects of this compound on both study species. This neonicotinoid pesticide also caused morphological malformations in birds exposed to 2.5–20 µg^65^, and fish exposed to 300 µg L^−1^ and 1000 µg L^−1^ of imidacloprid^33^, reflecting the more ample potential environmental impacts of this this insecticide.

Despite the alterations observed in the morphology of the tadpoles of both study species, only *L. luctator* presented changes in swimming activity after exposure to imidacloprid. In addition to the dietary changes resulting from the malformations of the oral structures and the intestine, a reduced food intake due to lethargy or increased energetic expenditure in individuals with hyperactivity may have contributed to the reduced growth and greater sensitivity of *L. luctator* to imidacloprid. Alterations of swimming activity have also been observed in other amphibians^13,35^, and fish^66^ exposed to imidacloprid, and a decrease in spontaneous locomotor activity has been recorded in rats^67^, possibly due to the neurobehavioral impacts of this pesticide^66^.

Genotoxic alterations were also observed in the *L. luctator* and *P. cuvieri* tadpoles exposed to imidacloprid in the present study. This genotoxic response has been associated with the increased production of reactive oxygen species by the pesticides, which promotes oxidative stress that inhibits the activity of the enzymes involved in DNA repair. This causes the formation of micronuclei and other nuclear abnormalities^68,69^. The frequency of all ENAs, particularly notched and lobed nuclei, increased considerably at imidacloprid concentrations of 30 µg L^−1^ and over, demonstrating the extreme genotoxic potential of this pesticide. Nuclear abnormalities are considered to be biomarkers of the impact of pesticides on amphibians^14,70,71^. As in the present study, notched nuclei were the most common abnormality in *Boana pulchella* tadpoles exposed to a pirimicarb-based compound^72^. Any external factor that affects cell proliferation, differentiation or apoptosis can produce embryotoxic or teratogenic effects, and may result in permanent congenital malformations, functional abnormalities or even the death of the individual﻿^73^.

Micronuclei, APs, NBs, and BCs all reached significant levels in both *L. luctator* and *P. cuvieri*, in particular at the highest imidacloprid concentrations. The formation of micronuclei is related to failures in mitotic division^74^ and may be triggered by the presence of nuclei with bubbles or cellular binucleation^75^, which results from the blockage of cytokinesis by abnormal cell division (reviewed by Benvindo-Souza et al.^76^). Apoptotic nuclei undergo nuclear disintegration without suffering any alteration of the cytoplasm^77^, which indicates cell death^76^ and is often associated with neurological disorders^78^. Exposure to higher concentrations of imidacloprid than those tested in the present study revealed genotoxic effects in the amphibians *Rana* sp. (exposed to 0.05 mg L^−1^, 0.5 mg L^−1^, 8 mg L^−1^, and 32 mg L^−1^ of imidacloprid)^37^, and *Boana pulchella* (15 mg L^−1^)^38^, as well as in the fish *Australoheros facetus* (100 µg L^−1^ and 1000 µg L^−1^)^79^, and *Prochilodus lineatus* (up to 1250 µg L^−1^)^80^. It is important to note that, due to the scarcity of data on the concentration of pesticides in the environment, the first studies were based on doses lower than those recommended for agricultural crops, although they were still high in comparison with the subsequent studies. The accumulation of data on both the amount of pesticides in the environment^40–42,81–83^ and their ecotoxicological effects on amphibians, has allowed the most recent studies to apply more realistic doses.

Based on the alterations in swimming activity and the morphological malformations and genotoxicity observed in the present study, we can conclude that 3 μg L^−1^ of imidacloprid can cause chronic effects for both *L. luctator* and *P. cuvieri*. At higher concentrations, both species are likely to present morphological malformations, and *L. luctator* may also exhibit reduced growth and altered swimming activity. At concentrations of over 3 μg L^−1^, *P. cuvieri* presents reduced growth, and the tadpoles of both species had genotoxic cell damage. The fact that the short-term assay used in the present study was sufficient to cause cytotoxic damage to the tadpole cells indicates just how potentially toxic this pesticide is to amphibians. Detectable concentrations of imidacloprid in water range from 0.001 to 320 μg L^−1^, although the mean maximum concentration in surface water is 18.65 μg L^−1^ (*n* = 21 studies; see Morrissey et al.^45^). This emphasizes the need for more research that focuses on limiting the concentrations of this pesticide at different trophic levels, and the importance of updating existing legislation to protect aquatic wildlife.

Although *L. luctator* and *P. cuvieri* belong to the same family (Leptodactylidae), are widely distributed in South America, and are adapted to a range of different habitats^10,84^, *L. luctator* was more sensitive to exposure to imidacloprid, and this is the first study to highlight this difference. A previous study^85^ also found differences in the sensitivity of the amphibians *Acris crepitans* and *Rana clamitans* to imidacloprid, which is an important consideration when selecting species as bioindicators, given that they need to be both sensitive to contaminants and abundant enough for systematic monitoring.

As tadpoles develop in a liquid medium, they are unable to escape exposure to contamination in aquatic environments, which may reduce their capacity to reach an adequate level of development to survive in the terrestrial environment. Neonicotinoid compounds such as imidacloprid are not only highly toxic but also persistent^86–88^, and impact distinct trophic levels in aquatic environments by reducing body mass, which alters the dynamics of the food chain, especially for the top-level consumers^89^. Given this, we emphasize the importance of the implementation of effective conservation measures, associated with the review or creation of specific legislation that will mediate the impact of pesticides on wild populations of anuran amphibians. The clear evidence of the toxic effects of imidacloprid on anuran amphibians and the extensive and unregulated agricultural use of other neonicotinoids in Brazil and worldwide highlights the need for further, more systematic research to better assess the risks of the use of these pesticides for anuran populations.

## Conclusion

We found that environmentally relevant concentrations of the neonicotinoid insecticide imidacloprid induced significant alterations in the development of the tadpoles of *L. luctator* and *P. cuvieri*. Significant morphological and genotoxic alterations were observed in both species, although *L. luctator* was more sensitive to the insecticide than *P. cuvieri*. Even the lowest tested concentration of the insecticide (3 μg L^−1^) was harmful to amphibians, a concentration 100 times lower than that permitted by environmental legislation in Brazil.

## Methods

### Tadpole species

Spawn of *L. luctator* and *P. cuvieri* were collected within 24 h of oviposition from non-agricultural land with no known use of pesticides in Erechim, Rio Grande do Sul state, Brazil (27°42′43.77″ S, 52°18′42.94″ W). The spawn was placed immediately in aquariums containing 15 L of dechlorinated water at the Ecology and Conservation Laboratory of the Erechim campus of the Federal University of Fronteira Sul. The eggs were raised under controlled conditions of temperature (24 ± 2 °C) and light (12/12 h light/dark photoperiod) until they reached development stage 25^90^. The water was monitored daily and presented the following parameters: pH = 7.5 ± 0.5, dissolved oxygen = 5.8 ± 0.4 mg L^−1^, turbidity =  < 5, conductivity = 649 ± 25 µS cm^−1^, hardness = 3.57 mg L^−1^, Na = 13.012 mg L^−1^, and Ni =  < 0.002 mg L^−1^. The tadpoles were fed daily with complete fish feed (Alcon Basic, Alcon) containing at least 45% crude protein and organic lettuce.

This study was approved by the Ethics Committee for the Use of Animals (CEUA) of the Fronteira Sul Federal University under protocols nº 8822130919 and nº 8742250320, and was authorized by the Chico Mendes Institute for Biodiversity Conservation (ICMBio) under license nº 72719. All methods were carried out in accordance with relevant guidelines and regulations, and as reported by the ARRIVE guidelines^91^.

### Experimental design and experimental conditions

The tadpoles in development stage 25 used in the tests had completely-formed oral structures, normal swimming activity, and had typical, homogeneous body length and mass. The tadpoles of *L. luctator* had a mean length of 13.25 ± 0.36 mm and body mass of 0.035 ± 0.008 g, while those of *P. cuvieri* had means of 16.60 mm ± 0.60 mm and 0.070 g ± 0.011 g.

These tadpoles were exposed to the insecticide in a static test over a standard period of 168 h (7 days) according to ASTM STP 1443^92^, during which, they were fed daily as described above. The tadpoles were exposed to five water treatments defined by the following nominal concentrations of imidacloprid (48% a.i., Imidacloprid Nortox, Nortox S/A, Arapongas, Brazil) added to the water of the aquarium: (i) 3 μg a.i. L^−1^, (ii) 30 μg a.i. L^−1^; (iii) 100 μg a.i. L^−1^; (iv) 200 μg a.i. L^−1^, and (v) 300 μg a.i. L^−1^, together with a control treatment, containing clean water only. The experiments were run with a randomized block design. Batches of 10 tadpoles were transferred to 500 mL glass containers, with each container being considered as an experimental unit. The assays were conducted in triplicate, with a total of 30 tadpoles per treatment. The physical–chemical characteristics of the water were the same as those used for the development of the tadpoles, with ammonia being measured daily (mean = 0.283 ± 0.038 mg L^−1^).

The pesticide concentrations were selected based on the imidacloprid value recorded in the surface water in Brazil (3 μg L^−1^)^40,41^ and in rice paddies in Vietnam (30 μg L^−1^)^42^, as well as the legal limit established in the Brazilian state of Rio Grande do Sul (300 μg L^−1^)^31^, and two intermediate concentrations. Merga and Van den Brink^93^ reported that the imidacloprid-based insecticide used in the present study remained at a constant concentration throughout their 96-h experiment. While the period of the present study was three days longer than this, and photolysis is known to degrade imidacloprid when luminosity or temperatures are high^94,95^, the conditions of the study were adequate to minimize either dissipation or degradation.

### Survival, swimming activity and body size and morphological malformations

Tadpole survival was verified every 24 h, when the number of live and dead tadpoles in each container was recorded. The dead tadpoles were removed from the containers. Swimming activity was also recorded every 24 h by qualitative observation, based on Rutkoski et al.^70^, with modifications. The tadpoles were stimulated gently with a glass rod and the response was recorded. For this, all the tadpoles in a given container were observed qualitatively at the same time by the same observer. Qualitative changes in behavior were assessed during the course of the experimental exposure by observing changes in the response of the tadpoles over a 1 min interval, using of a behavioral checklist, similar to that recommended for fish by ASTM E1711-12, to document the response of the animals. The activity of the tadpoles was classified as: (i) swimming activity equal to the control, (ii) lethargy (reduced swimming activity in comparison with the control), (iii) hyperactivity (increased swimming activity in comparison with the control), (iv) unresponsive (no movement), and (v) spasms (tremors and convulsions).

At the end of the assay period, the tadpoles were euthanized with lidocaine (5%) following the rules of the Brazilian National Council for Animal Control and Experimentation^96^. The total length (mm; snout to tail) and body mass (g) of these tadpoles were measured using digital calipers and a precision balance, respectively. Malformations of the oral structures (denticles or general morphology) and the intestine (edemas or general morphology) were evaluated according to Rutkoski et al.^70,97^. Digital images of the oral and intestine structures were obtained using a digital camera (P510, Nikon, Tokyo, Japan) and analyzed in comparison with the control, using a stereomicroscope (SZ51, Olympus, Tokyo, Japan).

### Micronucleus assay and other erythrocytic nuclear abnormalities

For genotoxic analysis, a drop of blood obtained from each of the 10 tadpoles selected randomly from each treatment was placed on slides and fixed and stained with Panotic Rapid stain (Laborclin Ltda, Brazil), according to the manufacturer's instructions. The slides were analyzed under an optical microscope with a 100 × lens (CX31, Olympus, Tokyo, Japan), with 1000 cells being examined from each individual. The cells were examined for the presence of erythrocyte nuclear abnormalities (ENAs), including micronuclei (MN). The micronuclei were analyzed following the protocol of Pérez-Iglesias et al.^98^, while the other six ENAs were analyzed according to Montalvão et al.^99^, being classified as apoptosis (AP), binucleated cells (BC), karyolysis (KA), lobed nuclei (LN), nuclear bubbles or buds (NB), and notched nuclei (NN).

### Statistical analyses

The normality and variance homogeneity of the data were confirmed using the Kolmogorov–Smirnov and Barlett tests, respectively. A one-way analysis of variance (ANOVA) was applied to the data on survival, body size, morphological malformations, swimming activity, MNs, and ENAs. Pairwise comparisons between each treatment and the control were based on Dunnett’s test (*p* < 0.05). The statistical analyses were performed in Statistic 8.0, and the graphs were produced in GraphPad Prism 7.0.

## Supplementary Information

Supplementary Tables.

## Data Availability

All data generated or analysed during this study are included in this published article (and its Supplementary Information files).

## References

[1] Karlsson O (2021). Pesticide-induced multigenerational effects on amphibian reproduction and metabolism. Sci. Total Environ..

[2] IUCN. *The IUCN Red List of Threatened Species.* Version 2021-3. https://www.iucnredlist.org (2022).

[3] Wake DB, Koo MS (2018). Amphibians. Curr. Biol..

[4] Campbell Grant EH, Miller DA, Muths E (2020). A synthesis of evidence of drivers of amphibian declines. Herpetologica.

[5] Green DM, Lannoo MJ, Lesbarrères D, Muths E (2020). Amphibian population declines: 30 years of progress in confronting a complex problem. Herpetologica.

[6] Mason R, Tennekes H, Sánchez-Bayo F, Jepsen PU (2013). Immune suppression by neonicotinoid insecticides at the root of global wildlife declines. J. Environ. Immunol. Toxicol..

[7] Adams E, Leeb C, Brühl CA (2021). Pesticide exposure affects reproductive capacity of common toads (*Bufo bufo*) in a viticultural landscape. Ecotoxicology.

[8] Frost DR (2021). Amphibian species of the world 6,1, an online reference. Electron. Datab..

[9] Eterovick, P. C., Souza, A. M. & Sazima, I. Anfíbios da Serra do Cipó [*Amphibians from the Serra do Cipó*]. http://herpeto.org/wp-content/uploads/2020/11/ANFIBIOS-DA-SERRA-DO-CIPO.pdf (PUCMINAS, 2020).

[10] Mijares, A., Rodrigues, M. T. & Baldo, D. *Physalaemus cuvieri* The IUCN Red List of Threatened Species, version 2014.3. http://www.iucnredlist.org (2010). Accessed 9 Jan 2015.

[11] de Sá FP, Zina J, Haddad CFB (2014). Reproductive dynamics of the Neotropical treefrog *Hypsiboas albopunctatus* (Anura, Hylidae). J. Herpetol..

[12] Herek JS (2020). Can environmental concentrations of glyphosate affect survival and cause malformation in amphibians? Effects from a glyphosate-based herbicide on *Physalaemus cuvieri* and *P. gracilis* (Anura: Leptodactylidae). Environ. Sci. Pollut. Res..

[13] Silva FL (2021). Swimming ability in tadpoles of *Physalaemus* cf. *cuvieri*, *Scinax x-signatus* and *Leptodactylus latrans* (Amphibia: Anura) exposed to the insecticide chlorpyrifos. Ecotoxicol. Environ. Contam..

[14] Pavan FA (2021). Morphological, behavioral and genotoxic effects of glyphosate and 2,4-D mixture in tadpoles of two native species of South American amphibians. Environ. Toxicol. Pharmacol..

[15] Simon-Delso N (2015). Systemic insecticides (Neonicotinoids and fipronil): Trends, uses, mode of action and metabolites. Environ. Sci. Pollut. Res..

[16] Pietrzak D, Kania J, Malina G, Kmiecik E, Wątor K (2019). Pesticides from the EU first and second watch lists in the water environment. Clean.

[17] IBAMA: Instituto Brasileiro do Meio Ambiente e dos Recursos Naturais Renováveis. Relatório de comercialização de agrotóxicos 2019 [*Brazilian Pesticide Marketing Report 2019*] https://www.ibama.gov.br/agrotoxicos/relatorios-de-comercializacao-de-agrotoxicos#boletinsanuais (2021).

[18] IBAMA: Instituto Brasileiro do Meio Ambiente e dos Recursos Naturais Renováveis. Vendas de ingredientes ativos por UF [*Active ingredient sales by UF in Brazil*]. http://ibama.gov.br/phocadownload/qualidadeambiental/relatorios/2019/Vendas_ingredientes_ativos_UF_2019.x (2021).

[19] IBAMA – Instituto Brasileiro do Meio Ambiente e dos Recursos Naturais Renováveis. Boletins anuais de produção, importação, exportação e vendas de agrotóxicos no Brasil [*Annual bulletins of production, import, export and sales of pesticides in Brazil*]. http://ibama.gov.br/index.php?option=com_content&view=article&id=594&Itemid=54 (2021).

[20] Pietrzak D, Kania J, Kmiecik E, Malina G, Wątor K (2020). Fate of selected neonicotinoid insecticides in soil–water systems: Current state of the art and knowledge gaps. Chemosphere.

[21] ANVISA: Agência Nacional de Vigilância Sanitária; Índice Monográfico I13. *Imidacloprido*. http://portal.anvisa.gov.br/documents/111215/117782/I13+%E2%80%93+Imidacloprido/9d08c7e5-8979-4ee9-b76c-1092899514d7 (2021).

[22] Kagabu S (2011). Discovery of imidacloprid and further developments from strategic molecular designs. J. Agric. Food Chem..

[23] Tomizawa M, Casida JE (2005). Neonicotinoid insecticide toxicology: Mechanisms of selective action. Annu. Rev. Pharmacol. Toxicol..

[24] Hashimoto F (2020). Occurrence of imidacloprid and its transformation product (imidacloprid-nitroguanidine) in rivers during an irrigating and soil puddling duration. Microchem. J..

[25] Hladik ML (2018). Year-round presence of neonicotinoid insecticides in tributaries to the Great Lakes, USA. Environ. Pollut..

[26] Jurado A, Walther M, Díaz-Cruz M (2019). Occurrence, fate and environmental risk assessment of the organic microcontaminants included in the Watch Lists set by EU Decisions 2015/495 and 2018/840 in the groundwater of Spain. Sci. Total Environ..

[27] Montagner CC (2019). Ten years-snapshot of the occurrence of emerging contaminants in drinking, surface and ground waters and wastewaters from São Paulo State, Brazil. J. Braz. Chem. Soc..

[28] CCME. Council of Ministers of the Environment. Canadian water quality guidelines for the protection of aquatic life. *Imidacloprid.* In Canadian water quality guidelines, Council of Ministers of the Environment. Winnipeg. https://ccme.ca/en/res/imidacloprid-en-canadian-water-quality-guidelines-for-the-protection-of-aquatic-life.pdf (2007).

[29] RIVM. Water quality standards for imidacloprid: Proposal for an update according to the Water Framework Directive in *National Institute for Public Health and the Environment*. https://www.rivm.nl/bibliotheek/rapporten/270006001.pdf (2014).

[30] PAN. Pesticide Action Network. *International Consolidated List of Banned Pesticides.* https://pan-international.org/pan-international-consolidated-list-of-banned-pesticides/ (2021).

[31] Brazil. *Secretaria Estadual da Saúde do Rio Grande do Sul.* Portaria SES RS nº 320, de 28 de abril de 2014. https://www.cevs.rs.gov.br/upload/arquivos/201705/11110603-portaria-agrotoxicos-n-320-de-28-de-abril-de-2014.pdf. (2014).

[32] Kobashi K (2017). Comparative ecotoxicity of imidacloprid and dinotefuran to aquatic insects in rice mesocosms. Ecotoxicol. Environ. Saf..

[33] Islam MA, Hossen MS, Sumon KA, Rahman MM (2019). Acute toxicity of imidacloprid on the developmental stages of common carp *Cyprinus carpio*. Toxicol. Environ. Health Sci..

[34] Pérez-Iglesias JM (2014). The genotoxic effects of the imidacloprid-based insecticide formulation Glacoxan Imida on Montevideo tree frog *Hypsiboas pulchellus* tadpoles (Anura, Hylidae). Ecotoxicol. Environ. Saf..

[35] Sievers M, Hale R, Swearer SE, Parris KM (2018). Contaminant mixtures interact to impair predator-avoidance behaviours and survival in a larval amphibian. Ecotoxicol. Environ. Saf..

[36] USEPA. United States Environmental Protection Agency. *Aquatic Life Benchmarks and Ecological Risk Assessments for Registered Pesticides.* https://www.epa.gov/pesticide-science-and-assessing-pesticide-risks/aquatic-life-benchmarks-and-ecological-risk. (2021).

[37] Feng S, Kong Z, Wang X, Zhao L, Peng P (2004). Acute toxicity and genotoxicity of two novel pesticides on amphibian, Rana N. Hallwell. Chemosphere.

[38] De Arcaute CR (2014). Genotoxicity evaluation of the insecticide imidacloprid on circulating blood cells of Montevideo tree frog *Hypsiboas pulchellus* tadpoles (Anura, Hylidae) by comet and micronucleus bioassays. Ecol. Indic..

[39] Nkontcheu DBK, Tchamadeu NN, Ngealekeleoh F, Nchase S (2017). Ecotoxicological effects of imidacloprid and lambda-cyhalothrin (insecticide) on tadpoles of the African common toad, *Amietophrynus regularis* (Reuss, 1833) (Amphibia: Bufonidae). Emerg. Sci. J..

[40] Bortoluzzi EC (2006). Contaminação de águas superficiais por agrotóxicos em função do uso do solo numa microbacia hidrográfica de Agudo, RS. Rev. Bras. Eng. Agric. Ambient..

[41] Bortoluzzi E. C. (2007). Investigation of the occurrence of pesticide residues in rural wells and surface water following application to tobacco. Quim. Nova.

[42] La N, Lamers M, Bannwarth M, Nguyen V. V., Streck T (2015). Imidacloprid concentrations in paddy rice fields in northern Vietnam: measurement and probabilistic modeling. Paddy Water Environ.

[43] Sweeney M. R., Thompson C. M., Popescu V. D. (2021). Sublethal, behavioral, and developmental effects of the neonicotinoid pesticide imidacloprid on larval wood frogs (Rana sylvatica). Environ. Toxicol. Chem..

[44] Gibbons C. A., Morrissey C, Mineau P (2015). A review of the direct and indirect effects of neonicotinoids and fipronil on vertebrate wildlife.. Environ. Sci. Pollut. Res..

[45] Morrissey C. A. (2015). Neonicotinoid contamination of global surface waters and associated risk to aquatic invertebrates: A review. Environ. Int..

[46] Stinson S A (2022). Agricultural surface water, imidacloprid, and chlorantraniliprole result in altered gene expression and receptor activation in *Pimephales promelas*.&nbsp;. Sci. Total Environ..

[47] DiGiacopo DG, Hua J (2020). Evaluating the fitness consequences of plasticity in tolerance to pesticides.. Ecol. Evol..

[48] Carlson BE, Langkilde T (2017). Body size variation in aquatic consumers causes pervasive community effects, independent of mean body size.&nbsp;. Ecol. Evol..

[49] Phung TX, Nascimento JCS, Novarr AJ, Wiens JJ (2017). Body size variation in aquatic consumers causes pervasive community effects, independent of mean body size.&nbsp;. Ecol. Evol..

[50] Beasley, V. R. Direct and indirect effects of environmental contaminants on amphibians. In *Reference Module in Earth Systems and Environmental Sciences*10.1016/b978-0-12-409548-9.11274-6 (Elsevier, 2020).

[51] Toledo LF, Sazima I, Haddad CFB (2011). Behavioural defences of anurans: An overview. Ethol. Ecol. Evol..

[52] Hartmann MT, Hartmann PA, Haddad CFB (2010). Reproductive modes and fecundity of an assemblage of anuran amphibians in the Atlantic rainforest, Brazil. Inheringia.

[53] Pupin NC, Gasparini JL, Bastos RP, Haddad CFB, Prado CPA (2010). Reproductive biology of an endemic *Physalaemus* of the Brazilian Atlantic forest, and the trade-off between clutch and egg size in terrestrial breeders of the *P. signifer* group. Herpetol. J..

[54] Pereira G, Maneyro R (2012). Size-fecundity relationships and reproductive investment in females of *Physalaemus riograndensis* Milstead, 1960 (Anura, Leiuperidae) in Uruguay. Herpetol. J..

[55] Tolledo J, Silva ET, Nunes-de-Almeida CHL, Toledo LF (2014). Anomalous tadpoles in a Brazilian oceanic archipelago: implications of oral anomalies on foraging behaviour, food intake and metamorphosis. Herpetol. J..

[56] Annibale FS (2020). Smooth, striated, or rough: how substrate textures affect the feeding performance of tadpoles with different oral morphologies. Zoomorphology.

[57] Venesky MD, Wassersug RJ, Parris MJ (2010). The impact of variation in labial tooth number on the feeding kinematics of tadpoles of southern leopard frog (*Lithobates sphenocephalus*). Copeia.

[58] Venesky MD (2013). Comparative feeding kinematics of tropical hylid tadpoles. J. Exp. Biol..

[59] Jones SKC, Munn AJ, Penman TD, Byrne PG (2015). Long-term changes in food availability mediate the effects of temperature on growth, development and survival in striped marsh frog larvae: implications for captive breeding programmes. Conserv. Physiol..

[60] Bach NC, Natale GS, Somoza GM, Ronco AE (2016). Effect on the growth and development and induction of abnormalities by a glyphosate commercial formulation and its active ingredient during two developmental stages of the South-American Creole frog, *Leptodactylus latrans*. Environ. Sci. Pollut. Res..

[61] Capellán E, Nicieza AG (2007). Non-equivalence of growth arrest induced by predation risk or food limitation: context-dependent compensatory growth in anuran tadpoles. J. Anim. Ecol..

[62] Chin AM, Hill DR, Aurora M, Spencer JR (2017). Morphogenesis and maturation of the embryonic and postnatal intestine. Semin. Cell Dev. Biol..

[63] Sun Y, Zhang J, Song W, Shan A (2018). Vitamin E alleviates phoxim-induced toxic effects on intestinal oxidative stress, barrier function, and morphological changes in rats. Environ. Sci. Pollut. Res..

[64] Ouellet, M. Amphibian deformities: current state of knowledge. In *Ecotoxicology of Amphibians and Reptiles *(eds Sparling, D. W. *et al*.) 617–661 (Society of Environmental Toxicology and Chemistry, 2000).

[65] Hussein M, Singh V (2016). Effect on chick embryos development after exposure to neonicotinoid insecticide imidacloprid. J. Anat. Soc. India.

[66] Crosby EB, Bailey JM, Oliveri AN, Levin ED (2015). Neurobehavioral impairments caused by developmental imidacloprid exposure in zebrafish. Neurotoxicol. Teratol..

[67] Lonare M (2014). Evaluation of imidacloprid-induced neurotoxicity in male rats: A protective effect of curcumin. Neurochem. Int..

[68] Žegura B, Lah TT, Filipič M (2004). The role of reactive oxygen species in microcystin-LR-induced DNA damage. Toxicology.

[69] Odetti LM, López González EC, Romito ML, Simoniello MF, Poletta GL (2020). Genotoxicity and oxidative stress in *Caiman latirostris* hatchlings exposed to pesticide formulations and their mixtures during incubation period. Ecotoxicol. Environ. Saf..

[70] Rutkoski CF (2020). Morphological and biochemical traits and mortality in *Physalaemus gracilis* (Anura: Leptodactylidae) tadpoles exposed to the insecticide chlorpyrifos. Chemosphere.

[71] Herek JS (2021). Genotoxic effects of glyphosate on *Physalaemus* tadpoles. Environ. Toxicol. Pharmacol..

[72] Natale GS (2018). Lethal and sublethal effects of the pirimicarb-based formulation Aficida® on *Boana pulchella* (Duméril and Bibron, 1841) tadpoles (Anura, Hylidae).

[73] Gilbert, S. F. *Developmental Biology*, 8th edn. (Sinauer Associates, 2006).

[74] Soto M, García-Santisteban I, Krenning L, Medema RH, Raaijmakers JA (2018). Chromosomes trapped in micronuclei are liable to segregation errors. J. Cell Sci..

[75] Crott J, Fenech M (2001). Preliminary study of the genotoxic potential of homocysteine in human lymphocytes in vitro. Mutagenesis.

[76] Benvindo-Souza M (2020). Micronucleus test in tadpole erythrocytes: Trends in studies and new paths. Chemosphere.

[77] Fenech M (2000). The in vitro micronucleus technique. The in vitro micronucleus technique. Mutat. Res..

[78] Podratz JL (2011). *Drosophila melanogaster*: A new model to study cisplatin-induced neurotoxicity. Neurobiol. Dis..

[79] Iturburu FG (2017). Uptake, distribution in different tissues, and genotoxicity of imidacloprid in the freshwater fish *Australoheros facetus*. Environ. Toxicol. Chem..

[80] Vieira CED, Pérez MR, Acayaba RDA, Raimundo CCM, Martinez CBR (2018). DNA damage and oxidative stress induced by imidacloprid exposure in different tissues of the neotropical fish *Prochilodus lineatus*. Chemosphere.

[81] Sanchéz-Bayo F, Goka K, Hayasaka D (2016). Contamination of the aquatic environment with neonicotinoids and its implication for ecosystems&nbsp;. Front. Environ. Sci..

[82] Wood T, Goulson D (2017). The environmental risks of neonicotinoid pesticides: a review of the evidence post-2013.. Environ. Sci. Pollut. Res..

[83] Craddock HA, Huang D, Turner PC, Quirós-Alcalá L, Payne-Sturges DC (2019). Trends in neonicotinoid pesticide residues in food and water in the United States, 1999–2015. Environ. Health.

[84] Heyer, R. *et al.**Leptodactylus latrans*. *IUCN Red List*10.2305/IUCN.UK.2010-2.RLTS.T57151A11592655.en (2010).

[85] Ade CM, Boone MD, Puglis HJ (2010). Effects of an insecticide and potential predators on green frogs and northern cricket frogs. J. Herpetol..

[86] Sarkar MA, Roy S, Kole RK, Chowdhury A (2001). Persistence and metabolism of imidacloprid in different soils of West Bengal. Pest Manag. Sci..

[87] Goulson D (2013). Review: An overview of the environmental risks posed by neonicotinoid insecticides. J. Appl. Ecol..

[88] Mineau, P. Neonic insecticides and invertebrate species endangerment. In *Reference Module in Earth Systems and Environmental Sciences*10.1016/B978-0-12-821139-7.00126-4 (2021).

[89] Yamamuro M (2019). Neonicotinoids disrupt aquatic food webs and decrease fishery yields. Science.

[90] Gosner KL (1960). A simplified table for staging anuran embryos and larvae with notes on identification. Herpetologica.

[91] Percie-du-Sert N (2020). The ARRIVE guidelines 2.0: Updated guidelines for reporting animal research. PLoS Biol..

[92] Herkovits, J. & Pérez-Coll, C. S. AMPHITOX: A customized set of toxicity tests employing amphibian embryos. Symposium on multiple stressor effects in relation to declining amphibian populations. In *Multiple Stressor Effects in Relation to Declining Amphibian Populations *(eds Linder, G. *et al.*) 46–60 (ASTM International STP 1443, 2003).

[93] Merga LB, Van den Brink PJ (2021). Ecological effects of imidacloprid on a tropical freshwater ecosystem and subsequent recovery dynamics.&nbsp. Sci. Total Environ..

[94] Bonmatin J.-M. (2015). Environmental fate and exposure; neonicotinoids and fipronil.. Environ. Sci. Pollut. Res..

[95] Sumon, K. A. *et al. *Effects of imidacloprid on the ecology of sub-tropical freshwater microcosms. *Environ. Pollut. ***236**, 432–441 (2018). 10.1016/j.envpol.2018.01.102 29414368

[96] CONCEA – Conselho Nacional de Controle e Experimentação Animal. Resolução normativa Nº 25, 29 de setembro de 2015. Guia Brasileiro de Produção, Manutenção ou Utilização de Animais para Atividades de Ensino ou Pesquisa Científica do Conselho Nacional de Controle e Experimentação Animal. http://www.mctic.gov.br/mctic/export/sites/institucional/institucional/concea/arquivos/legislacao/resolucoes_normativas/Resolucao-Normativa-CONCEA-n-27-de-23.10.2015-D.O.U.-de-27.10.2015-Secao-I-Pag.-10.pdf. (2015).

[97] Rutkoski CF (2018). Lethal and sublethal effects of the herbicide atrazine in the early stages of development of *Physalaemus gracilis* (Anura: Leptodactylidae).&nbsp;. Arch. Environ. Contam. Toxicol..

[98] Pérez-Iglesias JM, Soloneski S, Nikoloff N, Natale G, Larramendy ML (2015). Toxic and genotoxic effects of the imazethapyr-based herbicide formulation Pivot H® on montevideo tree frog *Hypsiboas pulchellus* tadpoles (Anura, Hylidae). Ecotoxicol. Environ. Saf..

[99] Montalvão MF (2017). The genotoxicity and cytotoxicity of tannery effluent in bullfrog (*Rana catesbeianus*). Chemosphere.

