# Supplementary material for: Toxicity and genotoxicity of imidacloprid in the tadpoles of Leptodactylus luctator and Physalaemus cuvieri (Anura: Leptodactylidae)
Source: Sci Rep. 2022 Jul 13;12:11926. doi: 10.1038/s41598-022-16039-z (PMC9279336; doi:10.1038/s41598-022-16039-z)
Supplement: Supplementary file 1 — Supplementary Tables. [file 41598_2022_16039_MOESM1_ESM.docx]

**Toxicity and genotoxicity of imidacloprid in the tadpoles of *Leptodactylus luctator* and *Physalaemus cuvieri* (Anura: Leptodactylidae)**

Caroline Garcia Samojeden^1^, Felipe André Pavan^1^, Camila Fátima Rutkoski^2^, Alexandre Folador^1^, Silvia Pricila Da Fré^1^, Caroline Müller^1*^, Paulo Afonso Hartmann^1^, Marilia Teresinha Hartmann^1^

**Supplementary Material**

**Table S1.** Percent survival, body length, and mass of Leptodactylus luctator and Physalaemus cuvieri tadpoles after 168 h of exposure to an imidacloprid-based insecticide.

| Species | Imidacloprid (μg L^-1^) | Survival (%) | Body length (mm) | Body mass (g) |
| --- | --- | --- | --- | --- |
| *Leptodactylus luctator* | 0 | 96.67 | 20.82 ± 0.30a | 0.086 ± 0.004a |
|  | 3 | 76.67 | 19.08 ± 0.28b | 0.046 ± 0.004b |
|  | 30 | 80.00 | 18.34 ± 0.30b | 0.037 ± 0.004b |
|  | 100 | 100.00 | 17.69 ± 0.30b | 0.044 ± 0.004b |
|  | 200 | 83.33 | 17.99 ± 0.30b | 0.048 ± 0.004b |
|  | 300 | 83.33 | 17.95 ± 0.31b | 0.032 ± 0.003b |
| *Physalaemus cuvieri* | 0 | 100.00 | 21.24 ± 0.25a | 0.100 ± 0.003a |
|  | 3 | 100.00 | 20.59 ± 0.28a | 0.095 ± 0.004a |
|  | 30 | 100.00 | 19.37 ± 0.30b | 0.080 ± 0.003b |
|  | 100 | 100.00 | 20.05 ± 0.26b | 0.092 ± 0.003b |
|  | 200 | 100.00 | 19.86 ± 0.23b | 0.094 ± 0.002b |
|  | 300 | 100.00 | 18.80 ± 0.31b | 0.072 ± 0.002b |

Data represent mean ± SEM (*n* = 3). Different letters indicate significantly different results according to the Dunnett´s test (*p* < 0.05).

**Table S2.** Number of individuals with occurrence of oral and intestinal malformations in Leptodactylus luctator and Physalaemus cuvieri tadpoles after 168 h of exposure to an imidacloprid-based insecticide.

| Species | Imidacloprid (μg L^-1^) | Number of examined tadpoles | | Tadpoles showing any malformation (%) | Tadpoles showing oral malformations (%) | Tadpoles showing intestinal malformations (%) |
| --- | --- | --- | --- | --- | --- | --- |
| *Leptodactylus luctator* | 0 | | 29 | 2(6.67)b | 0(0)b | 2(6.67)b |
|  | 3 | | 23 | 13(45.93)a | 13(45.93)a | 6(21.11)a |
|  | 30 | | 24 | 13(50.93)a | 13(50.93)a | 7(26.85)a |
|  | 100 | | 30 | 19(63.33)a | 19(63.33)a | 13(43.33)a |
|  | 200 | | 25 | 18(74.17)a | 18(74.17)a | 12(50.48)a |
|  | 300 | | 26 | 24(88.89)a | 24(88.89)a | 23(85.12)a |
| *Physalaemus cuvieri* | 0 | | 30 | 0(0.00)b | 0(0.00)b | 0(0.00)b |
|  | 3 | | 30 | 19(63.33)a | 19(63.33)a | 9(30.00)a |
|  | 30 | | 30 | 23(76.67)a | 23(76.67)a | 18(60.00)a |
|  | 100 | | 30 | 24(80.00)a | 23(76.67)a | 19(63.33)a |
|  | 200 | | 30 | 26(86.67)a | 26(86.67)a | 22(73.33)a |
|  | 300 | | 30 | 27(90.00)a | 27(90.00)a | 26(86.67)a |

Percentage of malformations are indicated in parenthesis. Different letters indicate significantly different results according to the Dunnett´s test (*p* < 0.05).

**Table S3.** Frequency (%) and number of Leptodactylus luctator tadpoles exposed to different concentrations of an imidacloprid-based insecticide showing alterations in swimming activity.

| Species | Imidacloprid  (μg L^-1^) | Lethargy | Hyperactivity | Spasms | Unresponsive |
| --- | --- | --- | --- | --- | --- |
| *Leptodactylus luctator* | 0 | 0(0)b | 0(0)b | 0(0)b | 0(0)b |
|  | 3 | 30(9)a | 0(0)b | 17(5)a | 23(7)a |
|  | 30 | 27(8)a | 27(8)a | 17(5)a | 13(4)a |
|  | 100 | 33(10)a | 27(8)a | 13(4)a | 13(4)a |
|  | 200 | 33(10)a | 13(4)a | 17(5)a | 10(3)a |
|  | 300 | 23(7)a | 30(9)a | 23(7)a | 23(7)a |
| *Physalaemus cuvieri* | 0 | 0(0)a | 0(0)a | 0(0)a | 0(0)a |
|  | 3 | 0(0)a | 0(0)a | 0(0)a | 0(0)a |
|  | 30 | 0(0)a | 0(0)a | 0(0)a | 0(0)a |
|  | 100 | 0(0)a | 0(0)a | 0(0)a | 0(0)a |
|  | 200 | 0(0)a | 0(0)a | 0(0)a | 0(0)a |
|  | 300 | 0(0)a | 0(0)a | 0(0)a | 0(0)a |

Number of tadpoles are indicated in parenthesis. Different letters indicate significantly different results according to the Dunnett´s test (*p* < 0.05).
